# Isolation and characterization of a novel podovirus which infects *burkholderia pseudomallei*

**DOI:** 10.1186/1743-422X-8-366

**Published:** 2011-07-26

**Authors:** Jiraporn Gatedee, Kanyanan Kritsiriwuthinan, Edouard E Galyov, Jinyu Shan, Elena Dubinina, Narin Intarak, Martha RJ Clokie, Sunee Korbsrisate

**Affiliations:** 1 Department of Immunology, Faculty of Medicine Siriraj Hospital, Mahidol University, Bangkok 10700, Thailand; 2 Faculty of Medical Technology, Rangsit University, Pathumthani 12000, Thailand; 3 Department of Infection, Immunity and Inflammation, Leicester Medical School, University of Leicester, Leicester LE1 9HN, UK

**Keywords:** *Burkholderia pseudomallei*, bacteriophage, *Podoviridae*

## Abstract

*Burkholderia pseudomallei *is a saprophytic soil bacterium and the etiological agent that causes melioidosis. It is naturally resistant to many antibiotics and therefore is difficult to treat. Bacteriophages may provide an alternative source of treatment. We have isolated and characterised the bacteriophage ΦBp-AMP1. The phage is a member of the *Podoviridae *family and has a genome size of ~ 45 Kb. Molecular data based on the gene which encodes for the phage tail tubular protein suggests that the phage is distinct from known phages but related to phages which infect *B. thailandensis *and *Ralstonia *spp. The phage ΦBp-AMP1 is the first *B. pseudomallei *podovirus to be isolated from the environment rather than being induced from a bacterial culture. It has a broad host range within *B. pseudomallei *and can infect all 11 strains that we tested it on but not related *Burkholderia *species. It is heat stable for 8 h at 50°C but not stable at 60°C. It may potentially be a useful tool to treat or diagnose *B. pseudomallei *infections as it can lyse several strains of clinical relevance.

## Findings

*Burkholderia pseudomallei *is a Gram-negative bacillus and the causative agent of melioidosis which is endemic in Southeast Asia and northern Australia [1]. The bacterium is a widely distributed environmental saprophyte commonly found in water and soil in the endemic areas. The bacterium is highly resilient to environmental stress and can survive in distilled water for months or even years [2]. It infects humans via inoculation through skin abrasions or via the inhalation route and causes either acute or chronic melioidosis. Acute infection is often manifested as septicemia, resulting in death within days of exposure. *B. pseudomallei *is resistant to many antibiotics including third-generation cephalosporins [3]. There is clearly a need for novel antimicrobials. One promising source of antimicrobials may come from bacteriophages which infect and kill specific bacterial cells. They are the most abundant living entities in the world, and play key roles in bacterial population dynamics and evolution [4]. Bacteriophages can be either temperate where they integrate into the host bacterial genomes, or virulent where they infect, multiply, and lyse their hosts without any initial integration period. The ability of such virulent phages to be used therapeutically has been well documented and reviewed [5,6].

Prophages have been shown to be abundant in *B. pseudomallei *genomes [7-9]. A recent study reported the abundance and diversity of prophages in *B. pseudomallei*, *B. thailandensis *and *B. mallei *genomes and suggested how they contribute to the phenotypic diversity in the *Burkholderia *species complex [8]. Pertinently to our study, the 37 phages identified in the *Burkholderia *genomes were either myoviruses, Mu-like viruses, or siphoviruses. Although one previous study reported the presence of a putative podovirus in the genome of *B. pseudomallei*, there are no published genetic studies to support this work [7]. Another recent study isolated six *B. pseudomallei *myoviruses from soil in Thailand, these were morphologically characterised to have lytic properties [10].

We report the isolation of a podovirus which is specific to *B. pseudomallei*. To isolate the phage, one hundred and fifty soil samples were screened. The samples were collected from rice fields within the Khon Kaen Province, Thailand. The soil samples were collected during the rainy season from a depth of around 10-20 cm. The ambient temperature in rice fields varies with season ranged from 23-35°C and in the summer time temperatures reach 40-45°C. Two grams of each soil sample was placed into 10 ml Luria-Bertani (LB) broth supplemented with 0.5 mM CaCl_2. _Each sample was mixed thoroughly, incubated at room temperature overnight, centrifuged at 4000 × g for 20 min and then passed through a 0.22 μm filter. Ten microlitre volumes were used in spot assays on *B. pseudomallei *strain K96243 grown on LB agar supplemented with 0.5 mM CaCl_2_. The phage was purified and kept as stock in SM-buffer with 50% glycerol at -70°C.

**Figure 1:**
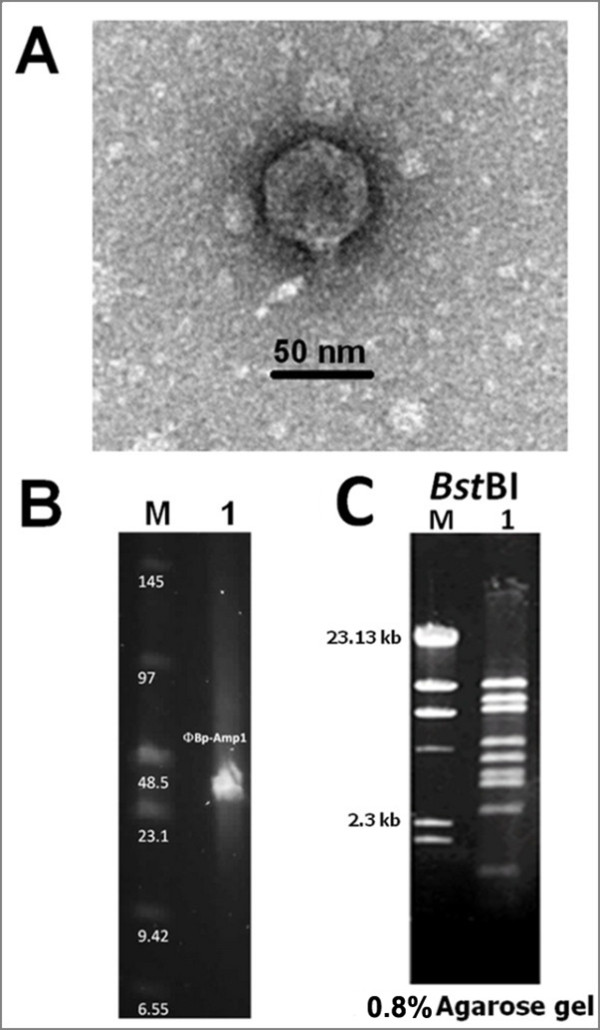
**Electron microscopic and DNA analysis of ФBp-AMP1.** (A) Transmission electron micrograph and ΦBp-AMP1 (B) The genome size of ΦBp-AMP1 determined by PFGE. (C) Restriction DNA pattern of ΦBp-AMP1 genomic DNA digested with *Bst*BI (lane1). Lane M, λ *Hind*III DNA marker.

The isolated phage was designated Bp-AMP1 and characterised using transmission electron microscopy (TEM). This analysis revealed that it belongs to the podovirus family as it has an icosahedral capsid with a diameter of ~ 45 nm and a characteristically small podovirus tail of ~20 nm tail (Figure 1A). Pulsed field gel electrophoresis (PFGE) was carried out as described previously [11]. This revealed that the genome size was consistent with a typical podovirus size of approximately 45 Kb (Figure 1B). Phage DNA was digested using the restriction enzyme *Bst*BI and a discrete DNA banding pattern was obtained (Figure 1C). The total size of the restriction fragments from this digestion amounted to 45.5 Kb and correlates with the PFGE estimate.

To investigate the host range of ΦBp-AMP1, plaque assays were performed on 10 *B. pseudomallei *strains isolated from distinct geographic locations, 6 *B. thailandensis *strains, 4 other *Burkholderia *spp., *Pseudomonas aeruginosa *and *Escherichia coli *strains (Table 1).

**Table 1:** Efficiency of ΦBp-AMP1 to form plaques on 10 *B. pseudomallei *and on other bacterial strains.

Bacteria	Strain	Source and location	Titres (pfu/ml)
*B. pseudomallei*	3073A	Clinical isolation, Thailand	(3.00 ± 0.41) × 10^7^
	576	Clinical isolation, Thailand	(2.70 ± 0.37) × 10^7^
	1710a	Clinical isolation, Thailand	(1.50 ± 0.15) × 10^6^
	BA18	Clinical isolation, Australia	(3.10 ± 0.33) × 10^5^
	MSHR42	Clinical isolation, Australia	(4.00 ± 0.44) × 10^5^
	MSHR287	Clinical isolation, Australia	(2.35 ± 0.24) × 10^6^
	MSHR668	Clinical isolation, Australia	10 ± 1.66
	E8	Soil, Thailand	(2.50 ± 0.20) × 10^4^
	E412	Soil, Thailand	(1.35 ± 0.11) × 10^6^
	MSHR491	Water, Australia	(5.00 ± 0.44) × 10^5^
*B.thailandensis*	D1	Soil, Thailand	-
	E28	Soil, Thailand	-
	E36	Soil, Thailand	-
	E68	Soil, Thailand	-
	E70	Soil, Thailand	-
	E94	Soil, Thailand	-
*B. multivorans*	LMG 16660	Clinical isolation, UK	-
*B. ubonensis*	DMST 866	Soil, Thailand	-
*B. vietnamensis*	LMG 6999	Clinical isolation, Vietnam	-
*B. cepacia*	ATCC 25416	-	-
*P. aeruginosa*	ATCC 27853	-	-
*E. coli*	ATCC 25922	-	-

*B. pseudomallei *K96243 was used to propagate the phages and the stock was used to infect the other strains. The stock gave 2.90 × 10^7 ^pfu/ml on K96243 and the titre on the other strains is given in the table.Value represent the mean pfu/ml ± S.E.M of triplicates, -, no plaque formation

ΦBp-AMP1 could infect all 11 *B. pseudomallei *strains however in general they were more efficient at infecting the Thai *B. pseudomallei *strains than the Australian strains. To do these efficiency assays, a known amount of phage stock derived from being propagated on K96243 was added to each host strain tested. This stock gave a titre of 2.90 × 10^7 ^pfu/ml when grown on the host strain. However, the titre was much lower on the Australian strains with the least good host being MSHR668 which only had a titre of 10 ± 1.66 pfu/ml. There are some exceptions to this and the Thai strain E8 was not an efficient host for the phage. It could not infect any of the tested 6 strains of *B. thailandensis *or the *B. multivorans, B. ubonensis, B. cepacia, B. vietnamiensis, P. aeruginosa *and *E. coli*. To the best of our knowledge, this is the first isolation of a *Podoviridae *family bacteriophage which specifically infects *B. pseudomallei*. Although *B. pseudomallei *myoviruses have been isolated previously, because myoviruses generally do not share any genes in common with podoviruses, ΦBp-AMP1 is likely to have a novel set of antimicrobial properties to those found in *B. pseudomallei *myoviruses.

**Figure 2:**
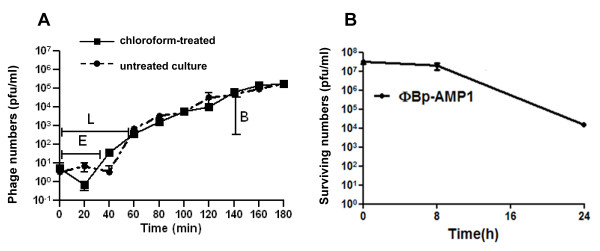
**Growth curve and thermal stability of ФBp-AMP1.** (A) One step growth curve of ФBp-AMP1 on *B. pseudomallei* strain K96243. Phage number (pfu/ml) in chloroform-treated (▀) and the untreated culture (●); E, eclipse period; L, latent period and B, burst size. (B) Thermal stability of ФBp-AMP1 at 50ºC. Values are the means of three independent experiments

In order to characterise the physiological characteristics of this phage, one-step growth curve experiments were performed as previously described [12]. Briefly, cultures of *B. pseudomallei *K96243 with ca. 1 × 10^8 ^bacteria were resuspended in 1 ml LB medium and incubated with ΦBp-AMP1 at a multiplicity of infection (MOI) 0.1. The mixture was incubated at 37°C for 10 min before centrifugation and resuspension in 10 ml of pre-warmed LB broth supplemented with 0.5 mM CaCl_2 _and incubated at 37°C. Five hundred microlitres of culture was taken at 20 min intervals over a period of 3 h and the number of phage particles was immediately determined using plaque assays. These data showed that the eclipse, latent period and burst size of ΦBp-AMP1 on *B. pseudomallei *K96243 at 37°C were 40 min, 60 min and 158 ± 54, respectively (Figure 2A). By comparison, the latent times of ΦBp-AMP1 was longer and burst size was smaller than the recently described *B. pseudomallei *phage ST79 which had a latent period of 15 min, and a burst size of 304 [10]. Although there appears to be a population of cells that are resistant to ΦBp-AMP1, this reflects the low titre of phages added to the culture. Bacteria from this culture were isolated and were still susceptible to phage infection and when higher MOI's were used in infection experiments total lysis was observed.

To investigate the thermal stability of the bacteriophages, a 10^7 ^pfu/ml phage stock was incubated at 50°C and 60°C for 0, 8 and 24 h. These temperatures are significantly higher than the ambient temperature of rice fields in Thailand. At intervals, the bacteriophage stock was titred. Figure 2B showed that ΦBp-AMP1 was stable for at least 8 h at 50°C but reduced from 10^7 ^pfu/ml to 10^4 ^pfu/ml after a further period of incubation from 8 h to 24 h. However, no bacteriophages were detected after 1 h incubation at 60°C (data not shown). This data indicates that ΦBp-AMP1 may be useful even in high temperature environments. The loss of phage viability at 60°C is a common observation, as seen for example in bacteriophages which infect *Lactobacillus delbrueckii *[13].

To obtain molecular data on this phage, we initially tried to amplify the DNA polymerase gene using designed primers from a subset of viruses belonging to the *Podoviridae *[14]. This was not successful probably due to a lack of sequence identity with known phages; we therefore randomly sequenced fragments of phage DNA. To do this, DNA was restricted using *Taq^α^*I and *Bst*BI and cloned into the plasmid pBluescript (Stratagene, La Jolla, CA, USA). Colonies were screened to identify those that contained suitable fragment sizes. A PsiBLAST (NCBI) search from a sequenced region identified a gene (EMBL:FR850500) which had a homologue with an ORF coding for a phage tail tubular protein B (TTPB) from a temperate *B. thailandensis *phage (GeneBank:ZP02468145.1). Fortuitously, this gene had been used as a phylogenetic marker in previous studies [15]. Therefore, a phylogenetic analysis was performed at the amino acid level using Molecular Evolutionary Genetics Analysis (MEGA) package version 4.1 (Beta) [16,17].

**Figure 3:**
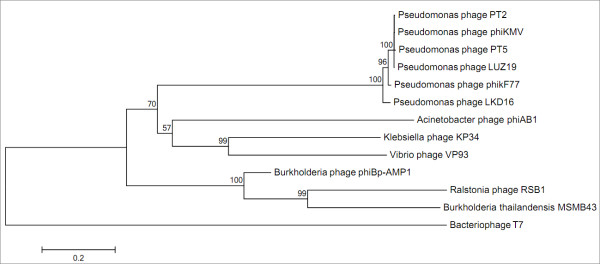
**Phylogenetic tree of ΦBp-AMP1 tail tubular protein B (TTPB) by the neighbour-joining method**. Branch lengths are indicated below the branches. Bootstrap values are indicated at the nodes. The scale bar represents the proportion of amino acid compared.

The tree in Figure 3 shows the results from a Neighbor joining analysis which was performed using a maximum composite likelihood model and a bootstrap analysis with 1, 000 replicates with bacteriophage T7 as an outgroup. The tree shows that ΦBp-AMP1 is clearly separated from known phages but is most closely related to the *B. thailandensis *MSMB43 prophage and the T7-like *Ralstonia *phage RSB1 (supported by a boots trap value of 100%). This is logical because *Ralstonia *is closely related to soil-born *Burkholderia *species [18]. Interestingly, there is no clear relationship between ΦBp-AMP1 and the characterised *Pseudomonas *phages. Analyses were also done using Parsimony and Maximum likelihood and all showed a similar tree topology.

In conclusion, we report the isolation of a podovirus which infects *B. pseudomallei*. The broad host-range and thermal stability of this phage suggests that it may have promise as a therapeutic agent. Further research is underway in our laboratories to characterise the genome of this bacteriophage and to establish its potential application for *B. pseudomallei *treatment.

## Competing interests

The authors declare that they have no competing interests.

## Authors' contributions

JG, KK and NI were responsible for soil samples collection. JG performed the phage biological characterisation, data analysis and drafted the manuscript. KK participated in the co-ordination of the study in Thailand. EEG, MRJC, and SK designed the study and interpreted the data. JS and ED carried out cloning, sequencing and phylogenetic analysis. SK and MRJC critically revised the manuscript. All authors have read and approved final manuscript.
